# “To Be or Not to Be” of a Polymer Nanogel—Unravelling the Relationship of Product Properties vs. Synthesis Conditions Governing the Radiation Crosslinking of Poly(acrylic acid) Using GPC/SEC—MALLS

**DOI:** 10.3390/ma16237467

**Published:** 2023-11-30

**Authors:** Sławomir Kadłubowski, Beata Paulina Rurarz, Joanna Raczkowska, Carlo Dessy, Piotr Ulański

**Affiliations:** 1 Institute of Applied Radiation Chemistry, Faculty of Chemistry, Lodz University of Technology, Wroblewskiego 15, 93-590 Lodz, Polandpiotr.ulanski@p.lodz.pl (P.U.); 2 Medical University of Lodz, Department of Cell Cultures and Genomic Analysis, Zeligowskiego 7/9, 90-752 Lodz, Poland; 3 Testa Analytical Solutions e.K., Sophienstr. 5, 12203 Berlin, Germany; cdessy@testa-analytical.com

**Keywords:** nanogels, poly(acrylic acid), radiation synthesis, gel permeation chromatography, multi-angle laser light scattering

## Abstract

In this paper, a state-of-the-art multi-detection gel permeation chromatography/size exclusion chromatography (GPC/SEC) system including multi-angle laser light scattering (MALLS) is applied to monitor radiation-induced synthesis of internally crosslinked nanostructures from poly(acrylic acid) (PAA). The aim is to demonstrate that this modern tool yields a more detailed picture of reaction mechanism and product structure than the techniques used to date. The prevailing intramolecular crosslinking narrows the molecular weight distribution from M_w_/M_n_ = 3.0 to 1.6 for internally crosslinked structures. A clear trend from over 0.7 to 0.5 in the Mark–Houwink exponent and a decrease in R_g_/R_h_ from 1.7 to 1.0 point to the formation of nanogels, more rigid and less permeable than the starting coils. Changes in the coil contraction factor (*g*′ = [*η*]*_irradiated_*/[*η*]*_linear_*) as a function of the radical density revealed the existence of two modes in intramolecular crosslinking, the initial one (up to 0.075 radicals per monomer unit) where the compactness of products changes strongly with progressing crosslinking and a second one where further compacting is suppressed by the lower flexibility of the partially crosslinked chain segments. This indicates a transition from soft, still internally crosslinkable nanogels to more rigid structures, less prone to further intramolecular loop formation. Our findings provide means for the tailored design of new PAA nanomaterials.

## 1. Introduction

Macroscopic polymer-based hydrogels and their nanosized analogues, nanogels, can be synthesized using a variety of methods. The approach studied in this work, that is, utilizing ionizing radiation to induce crosslinking in an additive-free aqueous polymer solution, is an interesting alternative both to the classical chemical synthesis of hydrogels [1,2,3,4,5,6,7,8,9] and to photochemical methods [10,11,12,13,14]. While the photochemical approach utilizes cheaper and more easily accessible equipment, it usually requires the presence of a photoinitiator (which may lead to the lower biocompatibility of products intended for medical use) and has some limitations when it comes to the penetration depth of UV light into the aqueous polymer solution. UV/laser light is also used for micro/nanostructure or nanocomposite formation in macroscopic hydrogels [15,16,17]. However, there are only few reports on the light-induced synthesis of polymer nanogels [18,19,20].

Ionizing radiation is particularly useful to synthesize polymer products for medicine. This is due to the fact that radiation processing is fast and easily controllable, requires no additional compounds such as initiators, catalysts or crosslinkers, hence obtained products can be readily chemically pure. Moreover, in some implemented technologies, such as production of hydrogel wound dressings based on radiation-crosslinked poly(N-vinylpyrrolidone) (PVP), radiation synthesis and sterilization can be accomplished in one step, thus reducing the number of required operations [21,22,23].

Radiation-synthesized nanogels, extensively tested as nanocarriers of drugs, genes and radioisotopes [24,25,26,27,28,29,30,31], can be brought as another excellent example, and various polymers, such as pullulan [32], PVP [33,34,35,36], chitosan and poly(acrylic acid) (PAA) [37], have been exploited with success for this purpose. PAA in particular is a very interesting polymer for biomedical applications, due to its biocompatibility and the contained -COOH groups, enabling the bioconjugation of selected ligands and “smart” drug delivery as a result of changes in the pH of the environment [38].

**Figure 1:**
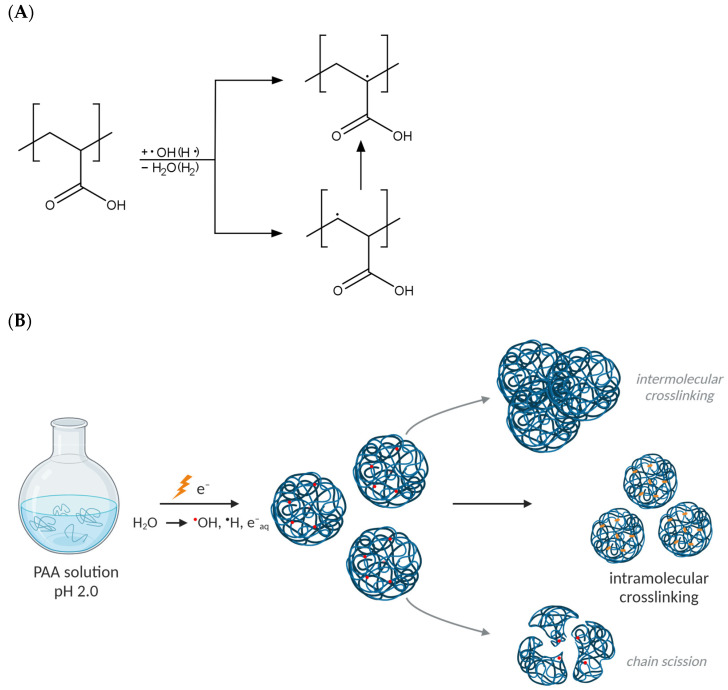
(**A**) Macroradicals formed upon the irradiation of dilute aqueous solutions of PAA. (**B**) Possible outcomes of radical reactions in PAA coils. Red dots denote radicals and orange dots denote crosslinks. Created with BioRender.com.

Upon the high-dose-rate irradiation of dilute aqueous polymer solutions with short pulses of fast electrons (the so-called pulse radiolysis regime), energy is absorbed both by macromolecules and water; however, only the latter effect is significant due to the immense prevalence of water molecules in the system. This in turn leads to water radiolysis (in a time interval lasting ca. 10^−12^ s) and the abundant generation of reactive species, such as hydroxyl radicals, hydrogen atoms and solvated electrons [39,40]. The yields of the particular products of water radiolysis depend on the irradiation conditions; however, at a low pH and in the presence of inert gases, such as argon, mostly hydroxyl radicals and hydrogen atoms play a role in the formation of polymer radicals (macroradicals) by hydrogen abstraction from the macromolecules [41,42]. The location and fate of macroradicals depend greatly on the chemical structure of the given polymer. In the case of PAA, α- and β-carboxyalkyl radicals are formed, as depicted in Figure 1A. Due to the fact that PAA is a weak polyelectrolyte, polymer chain dynamics and, as a consequence, the rates of macroradical reactions depend strongly on the pH level. In alkaline and neutral solutions, the negative charges of dissociated carboxylic groups cause repulsive forces to act between polymer segments, thus slowing down reactions between macroradicals and thus promoting single-radical-induced reactions, mainly chain scission [43]. To avoid this effect, and also to induce a coiled conformation of PAA macromolecules suitable for nanogel synthesis, the irradiation of PAA solutions in this work is performed at pH 2.0. Noteworthily, possible reactions of the radiation-generated macroradicals are recombination (leading to crosslinking, both inter- and intramolecular) and chain scission (Figure 1B). Intramolecular crosslinking is the most desired reaction that enables the formation of polymeric nanostructures—nanogels. It occurs when two radicals on the same polymer chain recombine and results in a virtually unchanged molecular weight followed by a decreased radius of gyration and intrinsic viscosity [44,45]. If changes in molecular weight are found, it indicates the contribution of either intermolecular crosslinking or chain scission in the case of increase or decrease in the molecular weight, respectively. Since, for intramolecular crosslinking, a number of radicals must be present simultaneously at one chain, specific reaction conditions are required, namely, subjecting polymer solutions to a short high-dose pulse (or a series of pulses) of ionizing radiation, allowing the generation of a high concentration of radicals within a short time. Therefore, these reactions were studied for PAA by irradiating its deoxygenated solutions at varying concentrations in the pulse radiolysis regime. To date, we examined the PAA of initial average molecular weight of ca. 0.5 MDa [46] and 1 MDa [47]—despite the conditions promoting intramolecular crosslinking, namely, low concentration and high dose rate, PAA radiation processing led to complex changes in the weight-average molecular weight (M_w_) and radius of gyration (R_g_), implying the combination of all three possible pathways of radical reactions. Therefore, we postulate that the understanding of the complicated synthetic processes leading to the formation of PAA nanogels could benefit from modern analytic modalities.

Among the methods useful for nanogels characterization, one can mention viscometry as well as light scattering, both static (SLS) and dynamic (DLS). These techniques allow for obtaining complementary data about the macromolecular structures dispersed in liquid media. However, with the emergence of combined techniques, which integrate multiple analytical modalities in one device, this field of research has profited considerably. Particularly, complementing the abovementioned techniques with chromatography can improve the analytic possibilities of nanogels. Gel permeation chromatography/size exclusion chromatography (GPC/SEC) provides information not only on the bulk/average properties, such as average molecular weight or viscosity, but also about the distribution of those properties.

Most GPC/SEC-aided research regarding polymers and polymeric nanogels is performed using GPC/SEC systems with differential refractive index (DRI) or differential pressure viscosity detectors. Those data acquisition modes can be used either purely qualitatively [34,48], or for quantitative applications, universal calibration is performed with well-characterized analytical standards, such as polystyrene [49] or poly(methyl methacrylate) [50] samples of different molar masses. However, molecular weights calculated based on the retention volumes of linear polymer standards do not yield correct molecular weight values for branched or internally crosslinked structures. It also does not fully exploit the GPC/SEC potential [49,51,52], and this led to the introduction of multi-angle laser light scattering detectors (MALLS) for absolute molecular weight measurements. Moreover, molecular weight distributions obtained with MALLS in combination with the data from conventional DRI and viscosity detectors can provide a much deeper insight into the nature of the structures obtained during polymer processing and into the process itself than ever before. For example, Zheng and coworkers, using the data derived from MALLS and DRI detectors, confirmed the core-shell structure of their poly(divinyl benzene-co-methyl methacrylate) nanogels [49]. Min et al. used the M_w_ values derived from MALLS and DRI to calculate the structural compactness of the hairy core-shell nanoparticles based on the methyl methacrylate—ethylene glycol dimethacrylate nanogels [53]. Inoue and coworkers explored intrinsic viscosity and M_w_ data to measure branching and thus prove that, upon progressing the crosslinking terpolymerization of multivinyl monomers, branched dendritic or nanogel-like structures were obtained [54]. However, to the best of our knowledge, there are no studies reporting a detailed analysis of the nanogel formation, down to the influence of the number of radicals per repeating unit on the crosslinking process. This can raise further questions about the nature of nanogels (or, more generally, the products of radiation-induced intramolecular crosslinking) with respect to the physicochemical properties of the structures obtained during the processing of poly(acrylic acid) in a deoxygenated diluted solution under high-dose-rate irradiation.

To address this challenge, we report the use of multi-detection GPC/SEC, instead of conventional GPC and batch SLS, to closely monitor the process of obtaining internally crosslinked nanostructures from linear poly(acrylic acid) of a relatively low molecular weight (M_w_ = 128 kDa). The development of structures from the dissolved linear polymer to the final product upon irradiation with fast electrons is followed using a state-of-the-art MALLS detection system for absolute molecular weight and viscosity detector for the evaluation of the intrinsic viscosity ([η]). Based on the changes in parameters, such as the Mark–Houwink equation’s ($\left[\eta \right]=KM_{\eta }^{\alpha }$) α parameter or the contraction factor g’ (calculated as the ratio between the intrinsic viscosities of the irradiated and unirradiated sample) [55], which in branched polymers is closely related to the number of side chains, we elaborate on the formation of a specific type of structures, such as non-linear chains formed by intermolecular and intramolecular crosslinking, as well as loosely or tightly internally crosslinked macromolecules. Based on our previous research, we also postulate that the fate of the polymer coils during pulse radiolysis processing is highly dependent on the concentration of the polymer in the solution [56]. A detailed investigation of the occurring changes should provide in-depth insights into the process of the radiation synthesis of polymer nanogels and possibly enable its further optimization.

## 2. Materials and Methods

### 2.1. Materials

Linear poly(acrylic acid) (PAA) with a nominal molecular weight of 250 kDa (35 wt.% solution in H_2_O, Cat. No. 416002-500ML, Lot STBG9347, Sigma Aldrich, Steinheim, Germany) was used without further purification. Polymer concentrations are expressed as mmol of monomer units/L (mM). Perchloric acid (HClO_4_, 70% in water) and sodium perchlorate (NaClO_4_·H_2_O) were procured from Sigma-Aldrich (Poznań, Poland). Anhydrous di-sodium hydrogen phosphate (Na_2_HPO_4_) and 1 M standard aqueous solution of sodium hydroxide (NaOH) were purchased from POCH S.A. (Gliwice, Poland). The samples were filtered with Minisart NML cellulose acetate syringe filters of 0.45 µm pore size (Cat. No. 16555, Sartorius Stedim Biotech GmbH, Göttingen, Germany). The eluent for GPC/SEC was filtered through filter paper (MN 617, Macherey-Nagel, Düren, Germany). Alanine pellets for dosimetry were purchased from Bruker (Billerica, MA, USA). Throughout all the experiments, TKA-Micropure (Niederelbert, Germany) filtered water was used.

### 2.2. Preparative Pulse Radiolysis of Poly(acrylic acid)

**Figure 2:**
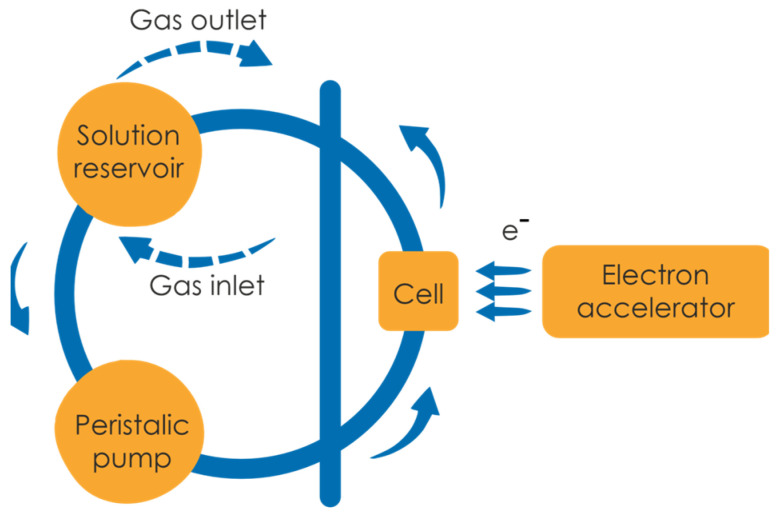
Closed-loop system used for the preparative pulse radiolysis of poly(acrylic acid).

Polymer irradiation was performed by a pulsed beam of high-energy electrons in a preparative pulse radiolysis regime, as described previously [47], with slight modifications. In short, 500 mL of a poly(acrylic acid) aqueous solution was prepared by dissolving polymer stock in water at 60 °C with moderate overnight stirring. The concentrations used in the experiments ranged from 10 mM to 25 mM. Prior to irradiation, the pH of the solution was set to 2.0 with HClO_4_ to protonate the carboxylic groups of polymer and thus compact the coils. Irradiation run for each solution was conducted at ambient temperature in a deoxygenated closed-loop system (circulation velocity 1 mL s^−1^), under continuous argon saturation. A schematic representation of the irradiation setup is depicted in Figure 2.

Short (4 μs) pulses of 6 MeV electrons at a frequency of 0.5 Hz were produced by the linear accelerator ELU-6 (Elektronika, Moscow, Russia), and the dose absorbed by a looping sample aliquot (0.7 mL in quartz cuvette) in a single pulse was ca. 1 kGy. The exact average dose per pulse was determined with alanine dosimetry (e-scan, Bruker (Billerica, MA, USA)) for each experiment. Each cycle consisted of a specific number of pulses, which ensured the irradiation of the whole circulating volume with a specific dose (as specified in the data graphs). Following the completed cycle, 50 mL of the sample irradiated with a specific dose was withdrawn from the solution reservoir. Subsequently, the system was re-deoxygenated for at least 10 min and the next cycle started. Five cycles of irradiation per run were performed, yielding 6 samples per single solution (including the non-irradiated control sample, collected prior to the first cycle of irradiation). After irradiation was completed, the samples were refrigerated until further analysis.

### 2.3. Gel Permeation Chromatography/Size Exclusion Chromatography (GPC/SEC)

To determine the molecular weight distribution, weight-average molecular weight (M_w_), radius of gyration (R_g_) and intrinsic viscosity ([η]), all samples were analyzed using gel permeation chromatography. The GPC/SEC system was provided by Testa Analytical Solutions (Berlin, Germany) and consisted of an isocratic pump, automatic injector, a set of 2 separation columns in a thermostatic column oven, as well as three detectors: multi-angle laser light scattering detector (MALLS) (Brookhaven Instruments Corporation, Holtsville, NY, USA), differential refractive index (DRI) and differential pressure viscosity detector, the last two combined in a single assembly with the common sample path (Testa Analytical Solutions, Berlin, Germany). Separation columns were supplied by PSS Polymer Standards Service GmbH (Mainz, Germany); Suprema Lux analytical Linear XL column (particle size 5.0 µm, pore size 100 Å, inner diameter 8 mm, length 300 mm, MALLS dedicated, suitable for neutral and anionic polymers) with Suprema Lux analytical SDV precolumn (to remove dust or large-sized aggregates) were used in all experiments. As the eluent, 0.1 M Na_2_HPO_4_ in water (pH 9.4) [57] was used after filtration with filter paper; the flow rate and temperature were maintained at 1 mL min^−1^ and 30 °C, respectively. The injection loop volume was 100 μL. The samples were injected to the instrument through a Minisart NML Cellulose acetate syringe filter of 0.45 µm pore size (Sartorius Stedim Biotech GMBH, Göttingen, Germany) to remove dust particles, as retrieved from the solution reservoir of the closed-loop irradiation system. Data were collected and processed with ParSEC GPC/SEC Software v5.72 (Brookhaven Instrument Corporation, Holtsville, New York, USA). All measurements were conducted in triplicate. All presented data points are the average values with the standard deviation error bars.

### 2.4. Dynamic Light Scattering (DLS)

The DLS technique was used to determine the hydrodynamic radius (R_h_) of the unirradiated and irradiated PAA solution. The analyses were performed using a standalone system consisting of an Innova 90C Ar ion laser (λ = 514.5 nm; Santa Clara, CA, USA), a BI-200SM goniometer (Brookhaven Instruments Corporation, Holtsville, NY, USA) and a TurboCorr Digital Autocorrelator (Brookhaven Instruments Corporation, Holtsville, NY, USA) at 25.0 ± 0.1 °C. Dynamic light scattering intensity was measured at 90°. The samples were prepared in an aqueous solution of 0.5 mol/L NaClO_4_, pH 10.0 (NaOH) and filtered through a syringe filter of 0.45 μm pore size (Minisart, Sartorius) before the analysis. All measurements were conducted in triplicate.

## 3. Results

Since the radiation synthesis of nanogels represents a very interesting alternative for classical chemistry approaches [58,59,60,61,62,63], there is no surprise that there is a growing interest in it. Since the first reports in late 1990s [44,45], there has been a growing number of studies on the radiation approach [24,25,32,34,64]. However, the process is still under investigation, and new methods have been employed to delve deeper into understanding the changes that occur in the polymers upon pulsed irradiation in a solution. Classically, the first-choice method used for the characterization of nanogels, including those based on PAA, is batch multi-angle static laser light scattering (MALLS) performed with a device equipped with a goniometer [47]. This allows the application of a broad spectrum of angles and, therefore, provides robust results. However, measurements performed with such a setup are tedious and time-consuming, and the obtained values, such as weight-average molecular weight, describe only the bulk, averaged properties of all macromolecules present in the solution. Therefore, in this paper, we employed a modern gel permeation chromatography system to improve our workflow and to obtain more insights into the mechanism of synthesis processes and into the structure of the synthesized samples. Our novel device was equipped with a MALLS detector operating at seven fixed angles as well as a combined differential refractive index and viscosity detector. Thus, we were able to not only evaluate the molecular weight of the polymer coils, but also investigate the changes in intrinsic viscosity at the same time. Moreover, we gained insights into the distribution of these values, which extends beyond what we were able to explore with classical batch MALLS or viscometry. The application of the multidetector (multi-angle laser light scattering (MALLS), differential pressure (viscosity) (DP), differential refractive index (DRI)) gel permeation chromatography for the analysis of nanogel synthesis also allowed us to obtain the following:

- Insights into the structure of the product using the Mark–Houwink equation evaluation;
- Early detection of structure changes as a function of the irradiation by close monitoring the alpha parameter in the Mark–Houwink equation;
- Determination of whether soft nanogels or more tightly packed particles are formed;
- Indication of the processes (e.g., intermolecular crosslinking, looping and branching) that are involved in nanogel formation.

Last but not least, a GPC/SEC-based setup, with simultaneous data acquisition at different angles, is simpler to use and operates significantly faster than the classical batch-mode MALLS.

**Figure 3:**
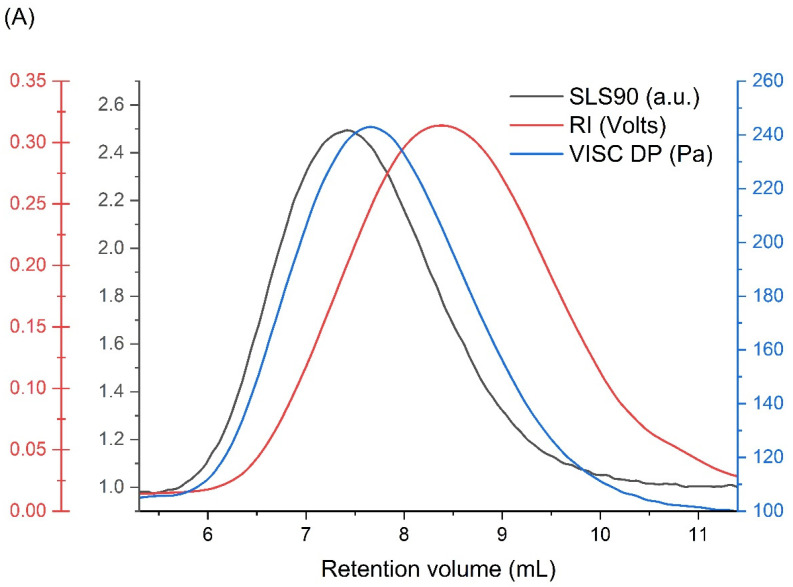

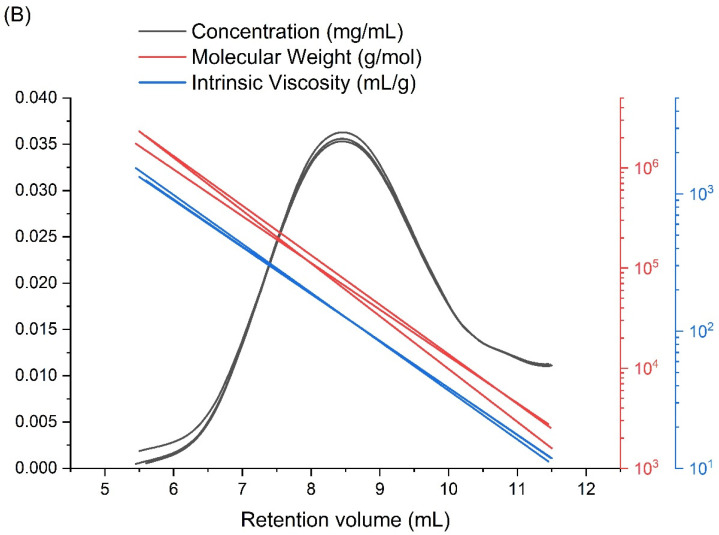
GPC/SEC analysis of the unirradiated poly(acrylic acid) aqueous solution: (**A**) chromatograms from each detector; and (**B**) molecular weights, viscosities and concentrations (based on information from MALLS, Viscometer Signal DP and RI detectors) as a function of the retention volume of three independent PAA solution injections. Eluent: 0.1 M Na_2_HPO_4_ aqueous solution (pH 9.4), [PAA] = 17.5 mM, temperature of 30 °C and flow rate of 1 mL min^−1^.

The examination of the ionizing radiation influence on the physicochemical properties of poly(acrylic acid) (PAA), i.e., the nanogel synthesis, was preceded by a detailed analysis of the unirradiated, linear polymer sample. Firstly, the applicability of the chosen column setup and measurement conditions (with respect to temperature, solvent and flow rate) as well as the repeatability of the GPC/SEC technique were studied for our samples of PAA. In Figure 3A, a representative chromatogram obtained for the unirradiated PAA solution (Figure 3A) and the distribution of molecular weights, viscosities and concentrations (based on information from MALLS, differential pressure of the viscometer and RI detectors) of three independent PAA solution injections (Figure 3B) are presented. One can see, in Figure 3B, that all chromatograms are almost identical, which shows the high reproducibility of the GPC/SEC analysis. Based on these data, the basic physicochemical properties of unirradiated PAA were calculated (Table 1).

Polymer irradiation was performed by a pulsed beam of high-energy electrons, as described in Section 2.2. After irradiation, the collected samples were analyzed (in triplicate) by gel permeation chromatography (Section 2.3).

**Figure 4:**
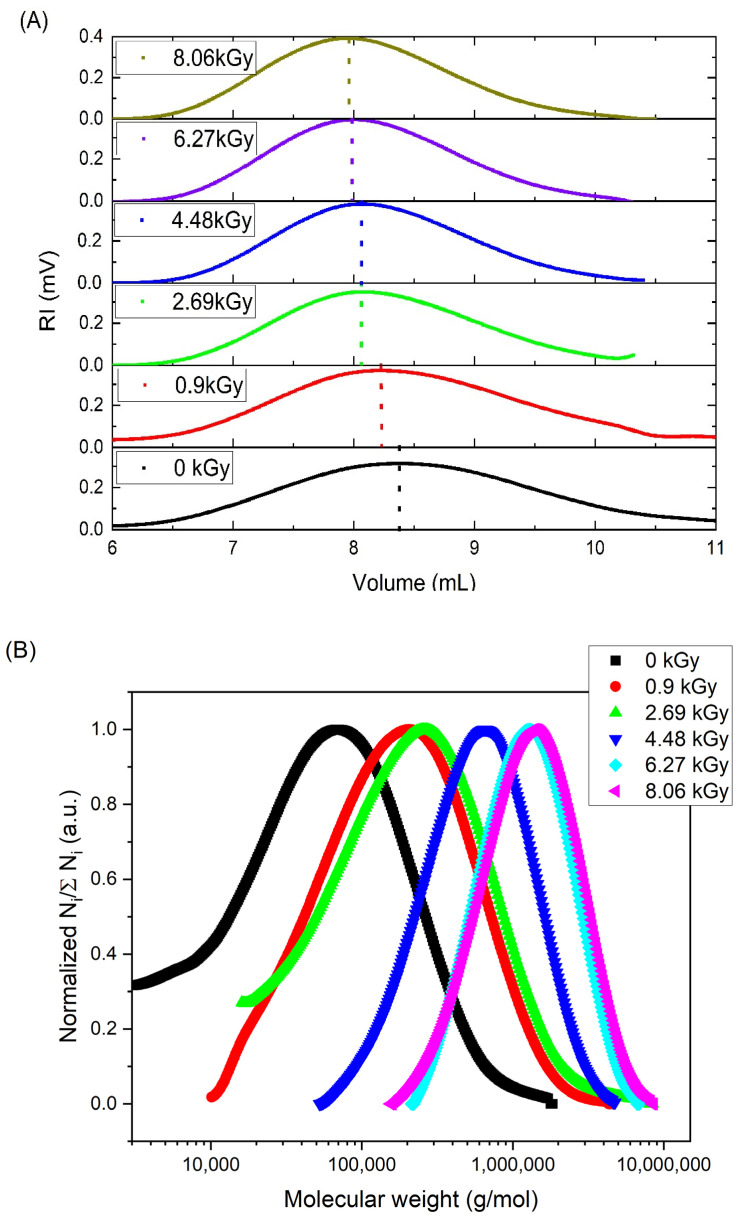
Changes in (**A**) the position of the concentration peak (RI detector) and (**B**) the normalized molecular weight distribution (defined as the ratio of the number of chains of a given molecular weight (N_i_) and the total number of chains (∑N_i_)) of the PAA solution irradiated with fast electrons using a linear accelerator (dose range 0–8.06 kGy)). Eluent: 0.1 M Na_2_HPO_4_ aqueous solution (pH 9.4), temperature of 30 °C and flow rate of 1 mL min^−1^. Irradiation parameters: dose per pulse 0.90 kGy, pulse duration of 4 µs and pulse frequency of 0.5 Hz. Solution properties during irradiation: [PAA] = 17.5 mM, pH 2.0 and saturated with Ar.

In Figure 4, the changes in (A) the position of the concentration peak (RI detector) as well as (B) the normalized molecular weight distribution of 17.5 mM PAA solution irradiated with fast electrons using the linear accelerator (dose range 0–8.06 kGy) are presented. A similar trend was observed for all the analyzed solutions (10–25 mM). One can see that the peak maximum shifted to the left side (smaller retention volumes) for higher absorbed doses. This indicates an increase in the hydrodynamic radius, and thus in most cases in the molecular weight. At the same time, the width of the peak became narrower, which, in other words, represents a decrease in the molecular weight distribution. This was also confirmed by the changes in M_w_/M_n_—as one can see, this parameter for the non-irradiated sample equaled 3.0, while upon irradiation for five cycles and 17.5 mM solution, it was reduced to 1.6. The molecular weight distribution calculated for the same set of samples presents both the narrowing of the width of the peak, i.e., a decrease in the molecular weight distribution and the accompanying increase in the average molecular weight.

**Figure 5:**
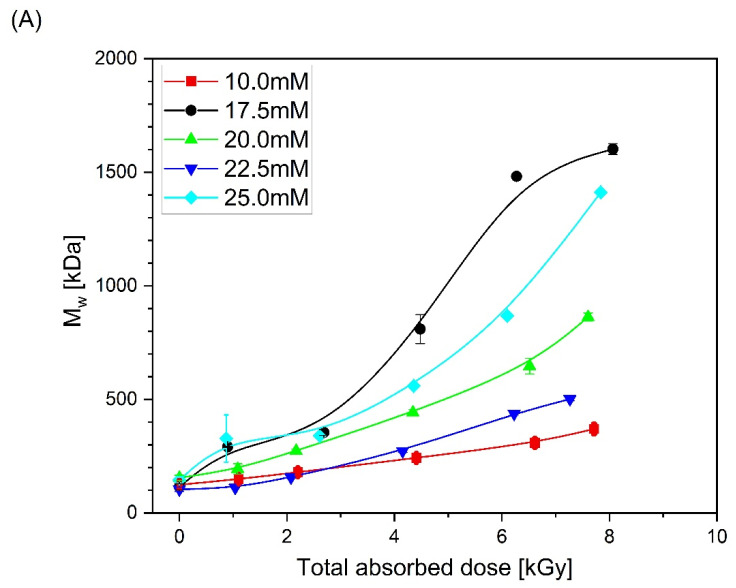

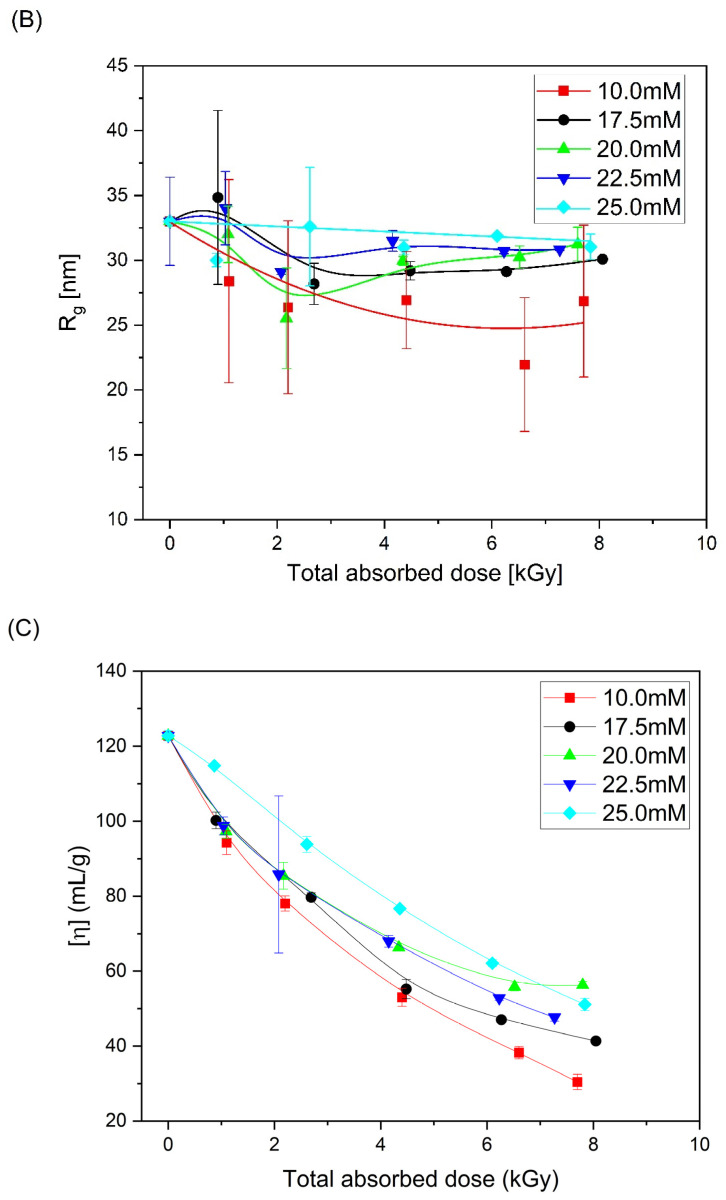
Changes in (**A**) the weight-average molecular weight, (**B**) radius of gyration and (**C**) intrinsic viscosity as a function of the total absorbed dose. Eluent: 0.1 M Na_2_HPO_4_ aqueous solution (pH 9.4), temperature of 30 °C and flow rate of 1 mL min^−1^. Irradiation parameters: dose per pulse 0.90 kGy, pulse duration of 4 µs, pulse frequency of 0.5 Hz and dose range of 0–8.06 kGy. Solution properties during irradiation: [PAA] = 10–25 mM (pH 2.0) and saturated with Ar. Lines were added as guidance.

Based on the obtained chromatograms, the weight-average molecular weight (M_w_), radius of gyration (R_g_) and intrinsic viscosity [η] of all products were calculated. Changes in these parameters as a function of total absorbed dose are presented in Figure 5. One can see that, together with the increase in molecular weight (regardless of PAA solution concentration), almost no change or slight decrease in the radius of gyration and a pronounced decrease in the intrinsic viscosity are observed, which is a typical feature that indicates intramolecular crosslinking as the dominating process and intermolecular crosslinking occurring side-by-side.

## 4. Discussion

Changes in the weight-average molecular weight and radius of gyration accompanying the radiation-induced synthesis of nanogels based on poly(acrylic acid) were reported previously by our group for PAA of a higher molecular weight (500–900 kDa) than that studied in this work [47]. It was found that the irradiation of dilute solutions of this polymer at a low pH with high-energy electrons ensures the simultaneous generation of carbon-centered radicals along the polymer chain. This results in, predominantly, intramolecular recombination, with a minor contribution of intermolecular crosslinks formation and chain scission. It should be noted that, as in the previous studies, all our experiments were conducted at a polymer concentration significantly lower than the coil overlap concentration; the latter, estimated for the irradiation conditions (pH 2.0) on the basis of the R_g_ of our PAA in that solvent [65], was ca. 135 mM.

The application of GPC/SEC with a triple detection system as described in Section 2.3 allowed us to investigate closely the changes in M_w_ and R_g_ (Figure 5A,B), but also the molecular weight distribution (Figure 4). The observed narrowing of the molecular weight distribution with the increase in the total absorbed dose can be attributed to the fact that the probability of the simultaneous formation of two or more radicals in a short chain is much lower than that in a long one; thus, short chains, bearing single radicals, cannot undergo intramolecular crosslinking (which does not change the molecular weight). Instead, their single radicals have to react with radicals located on other chains (intermolecular crosslinking), a process leading to the increase in the molecular weight. As a result, the molecular weight of the initially short chains grows rapidly. In contrast, long chains bear multiple radicals after being pulse-irradiated; in this case, the probability of a radical participating in intermolecular crosslinking is much lower, since most of the radicals react with their neighbors in the same chain, a process that does not lead to a molecular weight increase. Thus, the molecular weight increase in the initially long chains is not expected to be strong. Also, scission, if it occurs, is expected to take place randomly, and, if the initial M_w_/M_n_ is higher than 2 (which was the case in this paper), it is expected to contribute to making the distribution more narrow. Another effect that may contribute to the narrowing of the molecular weight distribution is that, when scission occurs, one of the resulting fragments bears a free radical and the other one a double bond. More double bonds may be formed by the disproportionation of radicals, which competes with crosslinking. As a result, mid-chain and terminal radicals can be added to double bonds. Therefore, the fragments formed by degradation may become again linked to other macromolecules, and thus they do not appear as short chains in GPC/SEC. It should be noted, however, that this description is strictly true for the initial stages of irradiation; as explained below, at high total absorbed doses, other factors, such as steric hindrance and chain stiffness, come into play.

**Figure 6:**
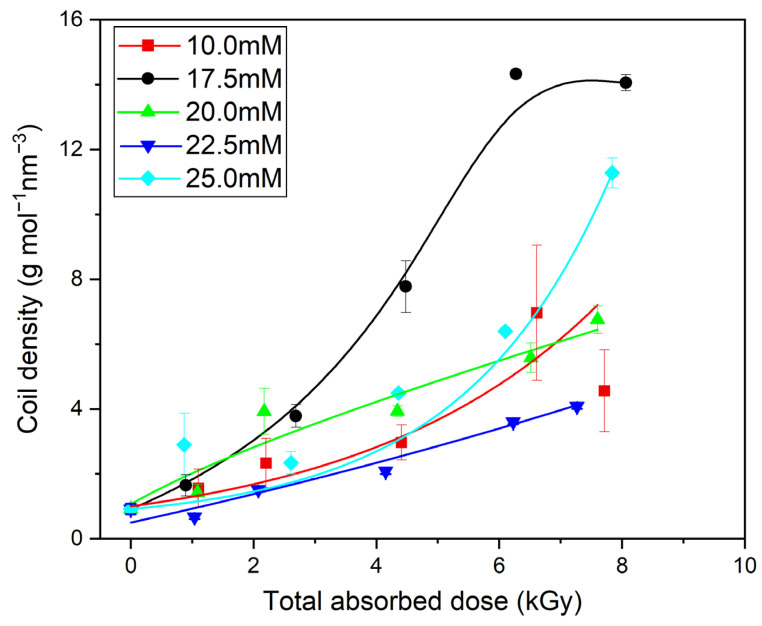
Changes in the coil density as a function of the total absorbed dose. Eluent: 0.1 M Na_2_HPO_4_ aqueous solution (pH 9.4), temperature of 30 °C and flow rate of 1 mL min^–1^. Irradiation parameters: dose per pulse 0.90 kGy, pulse duration of 4 µs, pulse frequency of 0.5 Hz and dose range of 0–8.06 kGy. Solution properties during irradiation: [PAA] = 10–25 mM (pH 2.0) and saturated with Ar. Lines were added as guidance.

In our previous works on high-molecular-weight PAA (500–900 kDa) [47], at the beginning of the irradiation process, especially at low concentrations, a slight decrease in the molecular weight caused by the chain scission was found. For the PAA studied in this paper, which had a relatively low starting M_w_ (ca. 130 kDa), we obtained no direct proof of the latter process (Figure 5A), albeit it very probably also occurs, but is masked by intermolecular crosslinking. At the same time, one can see that, similarly to polymers with longer chains, there was no increase or even decrease in the radius of gyration (Figure 5B). One should also notice that, in addition to the increase in the molecular weight, there was a strong decrease in the intrinsic viscosity (regardless of the concentration of the irradiated polymer). Altogether, it leads to the conclusion, also supported by Figure 6, that, in general, in line with the previous research, the irradiation of 130 kDa PAA with pulses of fast electrons leads to the formation of polymer nanogels of a higher coil density (defined as the ratio of the weight-average molecular weight and volume of the sphere with a radius equal to the radius of gyration) than the initial free coil formed by the parent linear macromolecule. An interesting observation regarding the changes in coil density as a function of the absorbed dose for solutions of different concentrations is that, for doses higher than 4 kGy, the densest irradiation product was obtained for the 17.5 mM solution. What is more, for the abovementioned solution, above 6 kGy, one can see the stabilization of the density of the obtained structures. Based on previously analyzed polymers, especially PAAs of higher molecular weight, it can be concluded that there is a strong competition between intra- and intermolecular recombination; degradation cannot be excluded either. For the 10 mM solution, a slight increase in the molecular weight was accompanied by the strongest, among all, decrease in the size. This could be assigned to the competition between chain scission and recombination (both intra- and intermolecular). Degradation is compensated by intermolecular crosslinking. The formation of intramolecular bonds between single-chain segments makes the structure less prone to losing fragments due to chain scission, and at the same time, due to loop formation, this leads to a decrease in the size and volume of the macromolecules [46]. For the concentration of 17.5 mM, there was a stronger influence and counterbalance of intermolecular crosslinking against degradation, and the strong influence of intramolecular crosslinking leads to the formation of a product with the highest coil (crosslink) density. At still higher concentrations, while all the trends (changes in M_w_, R_g_, viscosity and coil density) remained qualitatively the same with the increase in the dose, there was no clear trend in the magnitude of these effects as a function of the concentration. Nevertheless, data points for all concentrations converge when viscosity changes are correlated with radical density along the chains (see discussion below). A similar effect (increase in the molecular weight during irradiation of solutions of different concentrations) was observed previously during the synthesis of nanogels using the initial high-molecular-weight PAA [47] or PVP [66].

**Figure 7:**
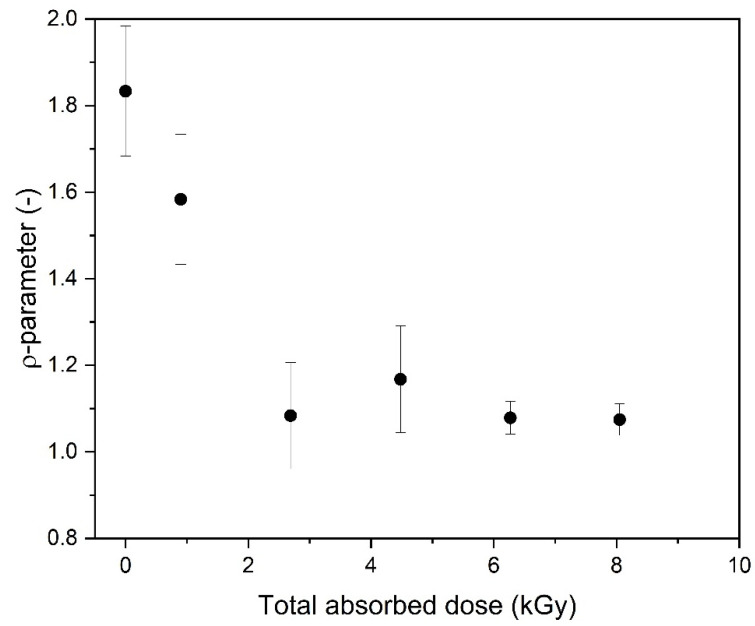
Changes in the ρ-parameter as a function of the total absorbed dose. The respective radii were measured in solvents with a high salt concentration, at a high pH (9–10). Determination of R_g_: GPC/SEC (eluent: 0.1 M Na_2_HPO_4_ aqueous solution, pH 9.4, temperature of 30 °C and flow rate of 1 mL min^−1^). Determination of R_h_: dynamic light scattering (solvent: 0.5 M NaClO_4_·H_2_O, pH 10.0). Irradiation parameters: dose per pulse of 0.90 kGy, pulse duration of 4 µs, pulse frequency of 0.5 Hz and dose range of 0–8.06 kGy. Solution properties during irradiation: [PAA] = 17.5 mM (pH 2.0) and saturated with Ar.

The formation of highly intramolecularly crosslinked macromolecules, i.e., nanogels, was also confirmed by the calculation of the ρ-parameter (ratio between the values of the radius of gyration (R_g_) and hydrodynamic radius (R_h_) obtained by means of static and dynamic light scattering, respectively). According to Burchard [55], it sensitively reflects the actual segment density as a result of the corresponding hydrodynamic interaction. Changes in the ρ-parameter for the 17.5 mM PAA solution as a function of the totally absorbed dose are presented in Figure 7. While in our case, the presented ρ values should be treated as approximate (R_g_ and R_h_ were determined in this paper in different solvents; see the Experimental Procedure Section), and the error bars are relatively large, it is clear that, while for the unirradiated sample the value (1.8) is characteristic for solutions of flexible, random coils of moderate polydispersity [55,67], the ρ-parameter becomes significantly lower for the irradiated samples, reaching a value of around 1, which is typical for highly branched or even hard, impermeable particles.

The dramatic change in the ρ parameter expected to reflect the topology of the product invokes questions when (in what conditions) soft nanogels are formed, what happens when irradiation is continued and what are the structure and properties of the product obtained using a high dose. Is the efficiency of intramolecular crosslinking constant or does it decrease when the product becomes highly crosslinked?

An answer can be obtained with a more detailed analysis of the GPC/SEC data, especially by the incorporation/utilization of light scattering and viscosity measurements.

**Figure 8:**
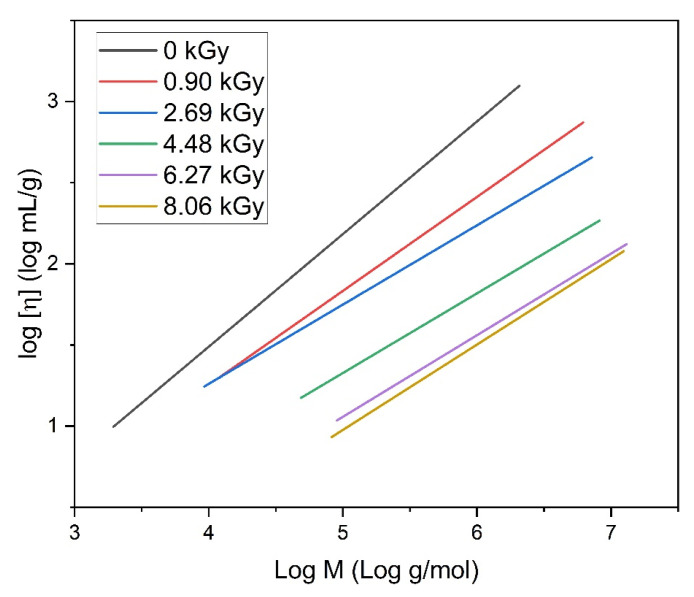
Light scattering and viscosity measurement data obtained for the PAA solution in a double logarithmic coordinate system corresponding to the Mark–Houwink equation. Eluent: 0.1 M Na_2_HPO_4_ aqueous solution (pH 9.4), temperature of 30 °C and flow rate of 1 mL min^−1^. Irradiation parameters: dose per pulse of 0.90 kGy, pulse duration of 4 µs, pulse frequency of 0.5 Hz and dose range of 0–8.06 kGy. Solution properties during irradiation: [PAA] = 17.5 mM (pH 2.0) and saturated with Ar.

In Figure 8, the light scattering and viscosity measurement data obtained for the representative PAA solution of 17.5 mM concentration in a double logarithmic coordinate system resulting from the Mark–Houwink equation ($\left[\eta \right]=K{\bar{M}}_{\eta }^{\alpha }$) are shown. This kind of representation allows us to evaluate the α-parameter from this equation. The value of the α-parameter determined by GPC/SEC for the non-irradiated solution is 0.71, which means that a 0.1 M Na_2_HPO_4_ aqueous solution is a thermodynamically good solvent for poly(acrylic acid). The values obtained for all irradiated solutions are presented in Table 2.

The α-parameter is a scalar value that is related to the stiffness of the polymer chains. In general, despite some scatter in the data, it is clear that, for all concentrations, the α values for the irradiated solutions are lower than the starting value of 0.71; for the lower concentrations, a clear decreasing trend is observed. For instance, for the 17.5 mM solution, with the increase in the absorbed dose, the α-parameter decreases to 0.5. This indicates an increase in segment–segment interactions, as this value is characteristic for the Flory theta solvent, pointing to a compact structure in contrast to an expanded, loose coil, corresponding to an α value of 0.7–0.8. An interesting phenomenon was observed for the 25 mM PAA solution. Regardless of irradiation, the α value is almost constant (around 0.6), which may be a result of polymer–polymer interactions in the solution of the highest concentration.

This shows that, from a thermodynamical point of view, by changing the polymer concentration and the absorbed dose, one can influence the crosslink density, which determines chain stiffness and interactions between the segments of macromolecules. But how to distinguish between different products of irradiation, i.e., loosely crosslinked nanogels and more tight structures, closer to spherical polymer nanoparticles with a low solvent permeability? An explanation was already provided through the calculation of the ρ-parameter (Figure 6). Gel permeation chromatography provides further insights into this problem.

**Figure 9:**
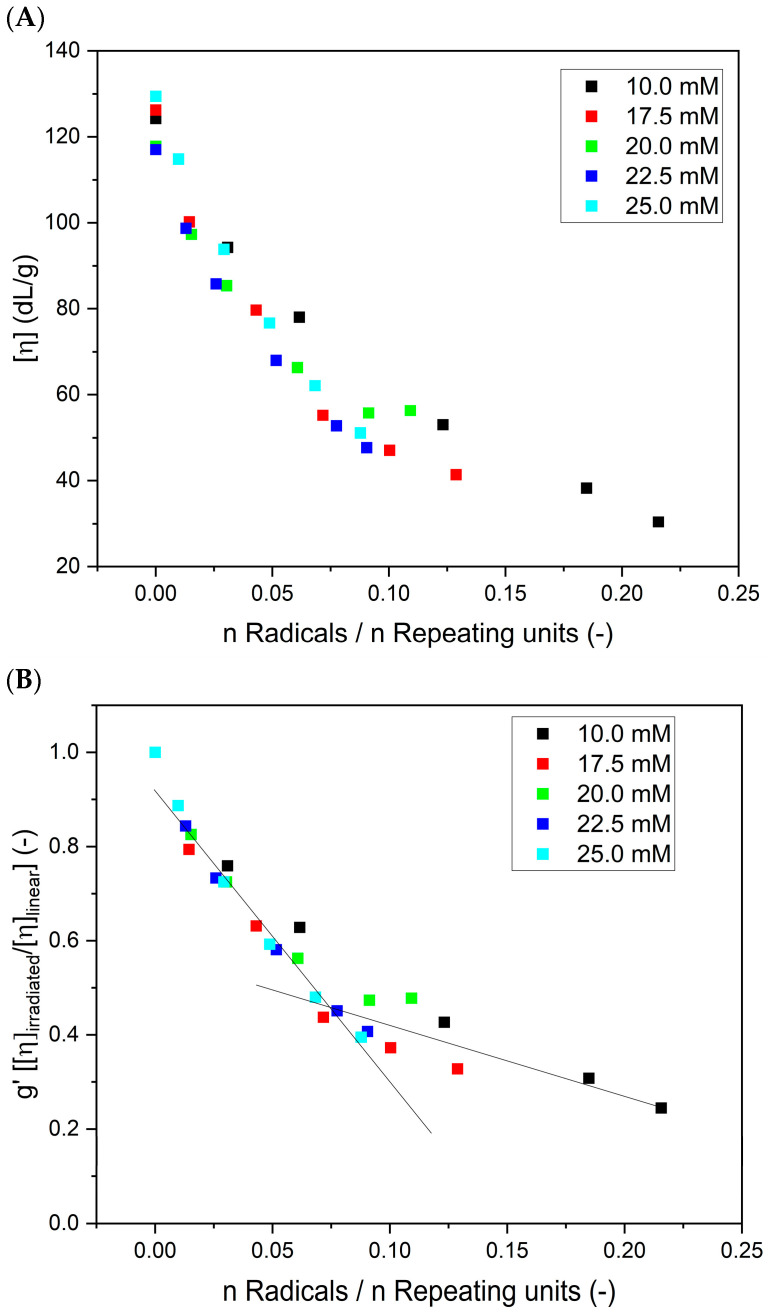

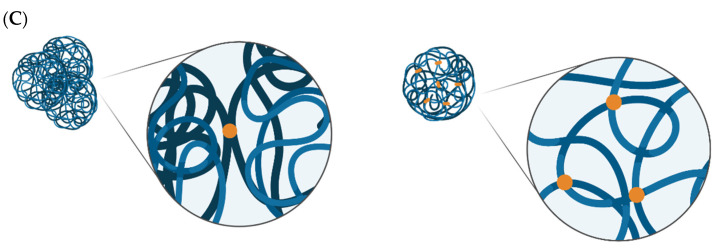
Changes in (**A**) the intrinsic viscosity and (**B**) contraction factor as a function of the ratio of the total number of radicals generated at a single coil to the number of repeating units. Eluent: 0.1 M Na_2_HPO_4_ aqueous solution (pH 9.4), temperature of 30 °C and flow rate of 1 mL min^−1^. Irradiation parameters: dose per pulse of 0.90 kGy, pulse duration of 4 µs, pulse frequency of 0.5 Hz and dose range of 0–8.06 kGy. Solution properties during irradiation: [PAA] = 10–25 mM (pH 2.0) and saturated with Ar. (**C**) Graphic representation of the differences between intermolecular crosslinking (left) and intramolecular crosslinking (looping) (right). Yellow dots denote newly formed bonds. Created with BioRender.com.

In Figure 9 (based on the data presented in Figure 5C), the changes in (A) intrinsic viscosities and (B) contraction factor ($g^{\prime }={\left[\eta \right]}_{irradiated}/{\left[\eta \right]}_{linear}$) as a function of the ratio between the total number of radicals generated at a single coil (calculated on the basis of the absorbed dose and polymer concentration; no correction for ^•^OH self-combination was applied) to the number of repeating units (calculated on the basis of molecular weight) are presented. One can see that the values of the intrinsic viscosity for different poly(acrylic acid) concentrations, but for an equal ratio between the number of radicals and the number of repeating units, are similar. An analogous behavior was observed through the Monte Carlo simulations of the intermolecular recombination of radicals [27]. That indicates that the products of a similar crosslink density can be obtained by the proper choice of the initial polymer concentration and applied radiation dose.

The results shown in Figure 9B demonstrate that, as expected, the more radicals are formed during irradiation per one monomer unit, the lower the contraction factor g’, indicating the increase in the compactness of the product. An interesting and previously not documented effect is the distinct change in the slope of the g’ parameter versus the number of radicals per monomer unit, which can be seen at n Radicals/n Repeating units around 0.075. This observation is in good correlation with the changes in the radius of gyration to hydrodynamic radius ratio (i.e., macromolecule structure) for the analyzed PAA solutions (Figure 7). When irradiation starts (low number of absorbed pulses and low number of radicals formed), the change in the g’ parameter is fast. This reflects the intramolecular crosslinking of free, linear chains. The formation of loops by the recombination of radicals located along the same chain makes the polymer coil more compact, which is reflected in a pronounced decrease in the coil dimensions, an effect typical for both chemical and radiation-induced intramolecular crosslinking [34,42,46,56,67]. It was also demonstrated by Monte Carlo simulations that the formation of a single loop (or a low number of loops) does not noticeably decrease the ability of the chain to form further loops [68]. This is explained by the fact that initially the loop formation causes two seemingly counteracting effects, which probably balance out each other—creating steric hindrance for the next recombination events between radical pairs, but also decreasing the average distance between the radicals within the macromolecule. Then, in Figure 9A,B, we see that, when a particular degree of internal crosslinking is reached, the further generation of radicals causes a markedly smaller effect on the size of the crosslinked macromolecule. This finding is in line with the levelling off in the coil density data for the 17.5 mM solution, shown in Figure 6 and Figure 9A. Very probably, when the chain becomes already highly crosslinked, with many loops formed, the steric hindrance effect begins to dominate over the reduced interradical distance effect; moreover, the chain becomes much less flexible, and this hinders the ability of radicals to find reaction partners on the same molecule. So, at that stage, when our product is no longer a very soft nanogel but becomes closer to a rigid, semi-permeable nanoparticle (albeit being still far from becoming a solid polymer nanoparticle), its ability to further internally crosslink becomes limited. On the other hand, we observe a further increase in the molecular weight (Figure 4 and Figure 5A); thus, at that stage, probably more radicals, unable to find a reaction partner within a relatively stiff nanoparticle, participate in crosslinking reactions occurring between the crosslinked chains. At the same time, some chain scission may also take place, contributing to the inability to form solid, completely impermeable nanoparticles. This indicates that, by controlling irradiation parameters (and effectively controlling the total number of radicals per monomer unit), we can influence the final structure of the product, reaching either relatively soft nanogels or more rigid, highly crosslinked (and not easily compressible anymore) structures. These observations confirm the applicability of multiple-detection HPLC-GPC for studying the particularities of non-linear polymer structures, which has been demonstrated before on branched and hyperbranched chains [69,70,71,72].

It is noteworthy that, despite the fact that our products indeed resemble hyperbranched structures, it is disputable whether they meet the classical definition of branched polymers. The character of hyperbranched coils is particularly pronounced when considering the properties and chromatographic separation behavior of our irradiated PAA, since in comparison to linear chains, we see clearly lowered hydrodynamic volume leading to a shift in the peaks on the GPC chromatograms. However, according to IUPAC, chain branching occurs when, in the reaction, there is a net increase in the number of reactive species [73]. The recombination of radicals, formed on the poly(acrylic acid) backbone during the irradiation, in essentially just the opposite—it leads to a reduction in reactive sites. These considerations additionally highlight the importance of understanding the origin and formation mechanisms of the analyzed samples in order to correctly interpret the obtained data. Our assumption, that hyperbranched structures are built once a given dose level at a given concentration is reached, is based again on the fact that the measured alpha parameter flattens out at a value of ca. 0.5, which points out to a dynamic equilibrium between intramolecular and intermolecular crosslinking as well as chain scission. Nanoparticles with still increased molecular density and increased chain stiffness, like ones that assumably would be built by continuous intramolecular crosslinking, are supposed to influence alpha toward a value close to null as the permeability of these nanoparticles must decrease with the increase in the density.

## 5. Conclusions

The nanoscale radiation engineering of polymers plays an emerging role in contemporary nanoscience. Fast, cost-effective, safe and environmentally friendly processes, initiated with reactive species derived from water radiolysis, yield materials with very promising properties. Still, we need novel analytical methods for a deeper understanding of those processes, to learn how to use them in our favor to obtain the desired products, tailored for the particular applications, such as medicine. The results presented in this work demonstrate the usefulness and advantages of applying a state-of-the-art multi-detection gel permeation chromatography/size exclusion chromatography (GPC/SEC) system, including multi-angle laser light scattering (MALLS), for studying the radiation-induced synthesis of polymer-based nanogels. Firstly, we showed that the results obtained by this technique for a model polymer, PAA, are in line with previous knowledge on the pulsed radiolysis of this polymer in dilute acidic solutions, intramolecular crosslinking being the main process leading to the formation of compact, internally crosslinked macromolecules, with intermolecular crosslinking and possibly chain scission as the side reactions. We successfully reproduced most of the main tendencies demonstrated before, i.e., increase in molecular weight with absorbed dose with no parallel rise in the radius of gyration (leading to the increase in coil density), and decrease in viscosity and in the R_g_/R_h_ ratio. While the accuracy of some of the obtained results may still need to be refined and some issues, such as the polymer concentration dependence of the M_w_ and coil density, need to be further clarified, the general picture emerging from M_w_, R_g_ and viscosity measurements is in line with what has been previously published for PAA of somewhat higher starting molecular weights than our sample. Moreover, applying multi-detection GPC/SEC allowed us to follow the reacting system in more detail than it was possible before, by studying new important properties, which have not been accessible to date. First of all, we could follow changes in molecular weight distribution. High-molecular-weight chains, bearing several radicals after an electron pulse, undergo mainly intramolecular crosslinking, with only limited increase in their average molecular weight. On the other hand, short chains, on which only single radicals are generated, undergo mainly intermolecular crosslinking, leading to a pronounced increase in molecular weight. As a result, the molecular weight distribution becomes narrower, the M_w_/M_n_ parameter being reduced (for 17.5 mM solution) from 3.0 to 1.6. The measurements of molecular weight and viscosity for each analyzed fraction of non-irradiated and irradiated samples allowed the following of the changes in the Mark–Houwink exponent, which is over 0.7 for linear PAA but drops to ca. 0.5 upon irradiation, indicating a substantial structural change from a free coil to much more rigid and less water-permeable, internally crosslinked particle. The detailed analysis of the coil contraction factor as a function of the average number of radicals per monomer unit revealed the existence of two modes of internal crosslinking. The first mode (for radical density up to ca. 0.075 radicals per monomer unit) is where the compactness of products changes relatively strongly with progressing crosslinking. It is followed by another mode, when further progress in making the chain more compact is weaker due to the lower flexibility of the (already partially crosslinked) chain segments. This observation indicates a transition, at a certain ratio of the number of radicals to the number of monomer units, from relatively soft and still internally crosslinkable nanogels to more rigid structures, less prone to undergo further internal loop formation. Summing up, the multi-detection GPC/SEC system with MALLS is a welcome and valuable new tool for studying complex processes of free-radical reactions in polymer solutions. It can facilitate the tailored design of new polymer nanomaterials with the desired physicochemical properties for future biomedical applications.

## Figures and Tables

**Table 1:** Basic physicochemical parameters of the unirradiated PAA, including the radius of gyration and intrinsic viscosity in 0.1 M di-sodium hydrogen phosphate buffer.

M_w_ (kDa)	M_n_ (kDa)	M_w_/M_n_	R_g_ (nm)	[η] (mL/g)
128 ± 21	42 ± 17	3.0	33 ± 4	123 ± 5

**Table 2:** The α-parameter of the Mark–Houwink equation of the PAA solution irradiated with fast electrons using a linear accelerator (dose range of 0–8.06 kGy). Eluent: 0.1 M Na_2_HPO_4_ aqueous solution (pH 9.4), temperature of 30 °C and flow rate of 1 mL min^−1^. Irradiation parameters: dose per pulse of ca. 1 kGy, pulse duration of 4 µs and pulse frequency of 0.5 Hz. Solution properties during irradiation: [PAA] = 10–25 mM (pH 2.0) and saturated with Ar.

	10.0 mM	17.5 mM	20.0 mM	22.5 mM	25.0 mM
1 cycle (ca. 1 kGy)	0.57	0.60	0.71	0.64	0.56
2 cycles (ca. 2 kGy)	0.59	0.53	0.53	0.57	0.63
3 cycles (ca. 4 kGy)	0.54	0.52	0.41	0.66	0.61
4 cycles (ca. 6 kGy)	0.45	0.50	0.44	0.61	0.61
5 cycles (ca. 8 kGy)	0.43	0.52	0.36	0.60	0.66

## Data Availability

Data are contained within the article.
